# The Effect of Psychological *Suzhi* on Suicide Ideation in Chinese Adolescents: The Mediating Role of Family Support and Friend Support

**DOI:** 10.3389/fpsyg.2020.632274

**Published:** 2021-02-11

**Authors:** Zhengguang Zhu, Wenchuan Tang, Guangzeng Liu, Dajun Zhang

**Affiliations:** ^1^Faculty of Psychology, Southwest University, Chongqing, China; ^2^School of Foreign Languages, Southwest University, Chongqing, China

**Keywords:** psychological *suzhi*, family support, friend support, suicide ideation, adolescents

## Abstract

In this study, we examined family support and friend support as potential mediators between psychological *suzhi* and suicide ideation in a sample of 1,369 Chinese adolescents (48. 1% men, 15.52 ± 1.76 years). The results showed that family support and friend support were found to adequately mediate the relationship between psychological *suzhi* and suicide ideation. In addition, the effect of psychological *suzhi* on adolescents' suicide ideation was stronger for family support than friend support. These findings demonstrated the key roles of psychological *suzhi*, family support, and friend support in reducing adolescents' suicide ideation. It enlightens us that we are supposed to improve adolescents' psychological *suzhi* and perceived social support (including family support and friend support) through many ways in order to better play its protective role in the future.

## Introduction

The 1990–2016 Global Suicide Disease Burden Analysis Report pointed out that, overall, ~800,000 people die of suicide every year in the world. Although the age-standardized death rate of suicide has dropped by 32.7% over the past 20 years, the phenomenon of suicide among teenagers has increased. It has become more and more common and has become a major global public health problem. Suicide has become the second leading cause of death among adolescents worldwide. Especially in the past 10 years, the suicide rate among people aged 10–14 years has almost tripled (Curtin and Heron, 2019); the rate of suicide among Chinese adolescents also did not decrease significantly (Liu Z. R. et al., 2017). Studies have pointed out that an individual's suicide will not only harm themselves and cause great grief to the family, but also induce friends or family members to increase suicide ideation or engage in suicidal behavior, which will have a great negative impact on society (Nanayakkara et al., 2013). Suicidal behavior includes suicide ideation, nonfatal suicide attempts, and fatal suicide (Silverman et al., 2007). Among them, suicide ideation refers to the loss of the desire to live, the start of thinking about plans and ways of suicide, and the notion and behavior that have not caused physical injury for the time being (Beck et al., 1973). From the perspective of suicide formation process, suicide ideation is the main link and inevitable stage of suicidal behavior, and it is also the most sensitive predictor (Miranda et al., 2014; Ni and Chen, 2016). Therefore, it is of great significance for the prevention and intervention of suicidal behavior of teenagers to find out the protective factors that influence suicide ideation and further reveal its influencing mechanism.

The stress-susceptibility model of suicide believes that suicide is a process jointly affected by stress factors (stress events), environmental factors (including family, social, cultural, and other factors), and individual qualities (including susceptibility, personality, cognition, etc.). The protective factors affecting suicidal behavior of adolescents mainly involve resilience, social support, and positive coping styles (Mann and John, 2002). Although there are abundant researches on the factors affecting suicide ideation, there is no research to examine if psychological *suzhi* affects the suicide ideation of adolescents during middle-school stage, or aiming at identifying a mechanism underlying such an effect (Johnson et al., 2010; Kristin and Sean, 2011; Evan et al., 2013). Therefore, this research aims to investigate the influence of middle-school students' psychological *suzhi* on suicide ideation from the perspective of psychological *suzhi*.

Psychological *suzhi* is a psychological concept with Chinese characteristics. It was first proposed under the background of Chinese vigorous promotion of quality education in the 1980s. Based on theoretical and empirical studies among Chinese students in the past 30 years, we defined it as a basic psychological quality. It has a derivative function related with individuals' developmental, adaptive, and creative behaviors and a multilevel self-organized system that involves steady implicit mental qualities and explicit adaptive behaviors (Zhang et al., 2011; Nie et al., 2020a). The psychological *suzhi* structure is based on the two-factor model, including general factors and special factors. The former highlights the basic characteristics of psychological *suzhi* and participates in all aspects of mental activities; the latter is specifically composed of three dimensions: cognitive quality, personality quality, and adaptation (Wu et al., 2017). Cognitive quality is the most basic component in the structure of psychological *suzhi*, which mainly refers to the mental quality of individuals when reflecting objective things, involving specific operations such as perception. Personality quality is the core of the structure of psychological *suzhi*, which reflects the individuals' personality psychology. Although it does not directly participate in the specific operations of cognition, it has motivation and regulation functions for the cognitive process. Adaptation mainly reflects the derivative function of psychological *suzhi*. It reflects the individuals' ability to continuously adapt to environmental changes during the process of interaction with the environment and to coordinate itself with the environment. It is a comprehensive manifestation of the integration of cognitive and individual factors into the individuals' external behavior (Nie et al., 2020b).

At present, the psychological factors concerned by suicide research mainly focus on variables such as self-esteem, depression, and hope, and there are few studies from the perspective of psychological *suzhi*. For a long time, depression has been regarded as an important risk factor for suicide. A large number of studies have shown that depression can significantly predict individual suicide ideation (Bradvik et al., 2008; Troister and Holden, 2013). At the same time, cognitive theory believes that the appearance of psychological feelings such as depression, loneliness, and suicide ideation is inseparable from self-knowledge (Beck, 1967). People with low self-esteem have insufficient problem-solving skills, and they are prone to extreme, absolute, and one-way judgments when encountering setbacks, which can lead to suicide ideation (Jibeen, 2017). In addition, studies have shown that the sense of hope is not only an effective negative predictor of suicide ideation, but also plays a significant role in regulating ruminant thinking and suicide ideation (O'Keefe and Wingate, 2013; Tucker et al., 2013), which means that hope can reduce the possibility of suicide ideation. Although no research has directly investigated the relationship between psychological *suzhi* and suicide ideation, many empirical studies have shown that adolescents' psychological *suzhi* can significantly positively affect self-esteem and hope (Liu G. Z. et al., 2017; Peng et al., 2020) and significantly negatively affect negative emotions such as depression and anxiety (Zhang et al., 2017). Therefore, we speculate that compared to specific mental health indicators such as self-esteem, depression, and hope, psychological *suzhi* as a comprehensive quality may play a more basic role, but the specific impact mechanism still needs to be further explored.

Perceived social support is the degree of emotional satisfaction that an individual feels subjectively supported and understood (Sarason et al., 1991). The buffer model of social support points out that social support can alleviate the negative effects of negative events and improve the individuals' physical and mental health (Cohen and Wills, 1985). Perceived social support and actual social support are not exactly the same. The former is psychological reality, and the latter is social reality. However, psychological reality is often more able to influence individuals' psychology and behavior; that is, perceived social support is also a potentially important protective factor (Brenning et al., 2015). There are abundant studies on perceived social support and suicide ideation. Some studies have found that compared with economic pressure and psychological resilience, perceived social support has a more significant influence on suicide ideation of adolescents (Lee et al., 2017), and other studies have pointed out that when adolescents have a higher level of perceived social support, it can significantly reduce the negative impact of bullying and reduce the possibility of suicidal ideation (Liu X. Q. et al., 2017). From the specific sources of social support, studies have pointed out that various family factors are risk factors for suicide among teenagers. Family function and parental warmth will have a significant impact on teenagers' suicide ideation (Sylvia, 2011; Li et al., 2016a). Some studies have also pointed out that after controlling for family structure and parental attachment, perceived peer support still has a strong predictive effect on suicide ideation (Li et al., 2016b). Perceived social support itself includes family support, friend support, and other support. Existing studies when investigating the relationship between social support and suicide ideation all consider social support as a whole or examining one aspect of support separately. Few studies examine the effects of family support and friend support separately, but this study aims to compare the two that have more significant effects. Therefore, we speculate that family support and friend support may simultaneously affect adolescents' suicide ideation, but we do not make assumptions about the specific impact mechanism.

Psychological *suzhi* and perceived social support are the potential factors affecting suicide ideation in adolescents, and there is also a close relation between them. The model of the relationship between psychological *suzhi* and mental health points out that psychological *suzhi* will affect the perception of external protective factors (such as social support), thereby affecting the individual's mental health (Zhang and Wang, 2012). As a core and basic psychological quality of adolescents, psychological *suzhi* has been shown to positively predict perceived social support, and can negatively influence social anxiety through the chain-like mediation of self-esteem and perceived social support (Zhang and Zhang, 2019). In other words, individuals with higher psychological *suzhi* have a higher level of perceived social support, and psychological *suzhi* can enhance the individual's mental health and social adaptation level by enhancing the perceived social support. And because it is mentioned above that perceived social support can significantly negatively affect adolescents' suicide ideation, we speculate that perceived social support may play a mediating role in the relationship between adolescents' psychological *suzhi* and suicide ideation.

In summary, this research will explore the relationship between psychological *suzhi*, perceived social support (family support, friend support), and suicide ideation in Chinese adolescents for the first time and subsequently attempt to examine internal mechanisms that account for these associations. Thus, we hypothesized that:

H1: Psychological *suzhi* is negatively related to suicide ideation.
H2: Psychological *suzhi* is positively related to friend support and family support.
H3: Friend support and family support would mediate the association between psychological *suzhi* and suicide ideation.

## Methods

### Participants and Procedures

Participants included 1,369 students recruited from six compulsory secondary education schools in eastern, central, and western China. We randomly selected 30 classes from 7th to 12th grade at six schools (272, 197, 204, 213, 284, and 199 participants, respectively). No significant differences were found in other demographic variables such as gender among schools, so these participants had a good sample representation. Two hundred thirty-six participants were in 7th grade, 211 were in 8th grade, 232 were in 9th grade, 237 were in 10th grade, 222 were in 11th grade, and 231 were in 12th grade. There were 658 (48.1%) boys and 711 (51.9%) girls. Participants were aged 11 to 20 years (mean = 15.52 ± 1.76 years).

The Research Ethics Committee of Chinese Southwest University approved this study. Before the start of the research, we contacted the administrators of the relevant schools, obtained permission for the questionnaire test, and obtained informed consent from the students and parents at the parents' meeting. At the beginning of the test, the researcher introduced the research objectives and procedures to the participants. Then, the participants filled out the questionnaire under the supervision of the researcher and completed it within the specified time. All participants were tested during regular school hours in their classrooms.

### Measures

#### Psychological *Suzhi*

Psychological *suzhi* was measured by the Psychological *Suzhi* Questionnaire for Middle School Students (Hu et al., 2017). This scale contains 24 items. It measures psychological *suzhi* from three dimensions including cognitive quality (e.g., “I always have explicit pathways when doing exercises”), individuality (e.g., “I always press myself to complete what I should do”), and adaptability (e.g., “I am a popular person”). Please see full details of the questionnaire in the Supplementary Materials. Each item was rated on a 5-point scale (1 = totally disagree, 5 = totally agree), with higher scores indicating greater psychological *suzhi*. In this study, Cronbach α of the total scale was 0.92 and ranged from 0.79 to 0.84 for the subscales.

#### Perceived Social Support

Perceived social support was measured by the Multidimensional Scale of Perceived Social Support (Zimet et al., 1988). This scale contains 12 items, which were answered using a 7-point rating scale from 1 (strongly disagree) to 7 (strongly agree), with higher scores indicating greater perceived social support. It measures perceived support from three aspects including family, friends, and significant others. This scale is suitable for use with adolescents in the Chinese school environment, and the reliability of the whole scale is excellent (Chen et al., 2016). For research purposes, this study selected only eight items to evaluate family support and friend support and scored the two parts separately. In this study, Cronbach α values of family support and friend support were 0.83 and 0.86, respectively.

#### Suicide Ideation

Suicide ideation was measured by the Positive and Negative Suicide Ideation (PANSI; Osman et al., 1998). This scale contained a six-item subscale of positive suicide ideation and an eight-item subscale of negative suicide ideation. Positive suicide ideation was scored in reverse, and the total score of negative suicide ideation was added to obtain the total score of suicide ideation. Higher scores on the PANSI indicate stronger suicide ideation. Each item was rated on a 5-point scale (1 = none of the time, 5 = most of the time). PANSI has been shown to have high reliability and validity in the Chinese school environment (Wang et al., 2011). Cronbach α values of positive suicide ideation and negative suicide ideation were 0.82 and 0.93 respectively, and 0.89 for the total scale in this study.

#### Analytic Strategy

The collected data were analyzed using SPSS Statistics version 24.0 and Mplus 7. First, descriptive analysis was conducted to calculate the mean and standard deviation for each variable. Next, correlational analyses were conducted to examine whether psychological *suzhi* was associated with the friend support, family support, and suicide ideation in the expected directions. And then, the measurement model's goodness of fit was evaluated. The following indexes were used to assess goodness of fit for the measurement model: comparative fit index (CFI), Tucker-Lewis index (TLI), root mean square error of approximation (RMSEA), and standardized root mean square residual (SRMR). CFI and TLI values of ≥0.90, SRMR values of ≤0.08, and RMSEA values of ≤0.08 indicated good model fit (Hu and Bentler, 1999). Finally, multiple mediation analyses were conducted to examine the mediating roles of family support and friend support in the relationship between psychological *suzhi* and suicide ideation by using SEM. Following existing studies, age and gender were included as control variables. In this study, we obtained 5,000 bootstrap resamples and used them to determine the 95% confidence intervals (CIs) of the indirect effects. If the CI did not include zero, then the indirect effect was significant at *p* = 0.05 (Preacher and Hayes, 2008).

## Results

### Descriptive Statistics Among the Variables

Table 1 displays the means and standard deviations, and the correlations between psychological *suzhi*, friend support, family support, and suicide ideation. Psychological *suzhi* was significantly positively correlated with friend support and family support. Both of them were significantly negatively correlated with suicide ideation.

**Table 1:** Descriptive statistics and bivariate correlations between study variables.

**Variables**	**1**	**2**	**3**	**4**
1. Psychological *suzhi*	–			
2. Friend support	0.29***	–		
3. Family support	0.34***	0.48***	–	
4. Suicide ideation	−0.38***	−0.29***	−0.39***	–
Mean	86.05	19.36	19.65	25.73
Standard deviation	13.52	5.04	5.05	7.72

*** *p < 0.001*.

### Mediation Analysis

As two potential parallel mediators, friend support and family support were entered into a mediation model to examine whether they mediated the link between psychological *suzhi* and suicide ideation in Chinese adolescents. The test of the hypothesized mediator model showed a good date fit (χ^2^ = 863.45, *df* = 84; CFI = 0.91, TLI = 0.88, RMSEA = 0.08, SRMR = 0.07).

**Figure 1:**
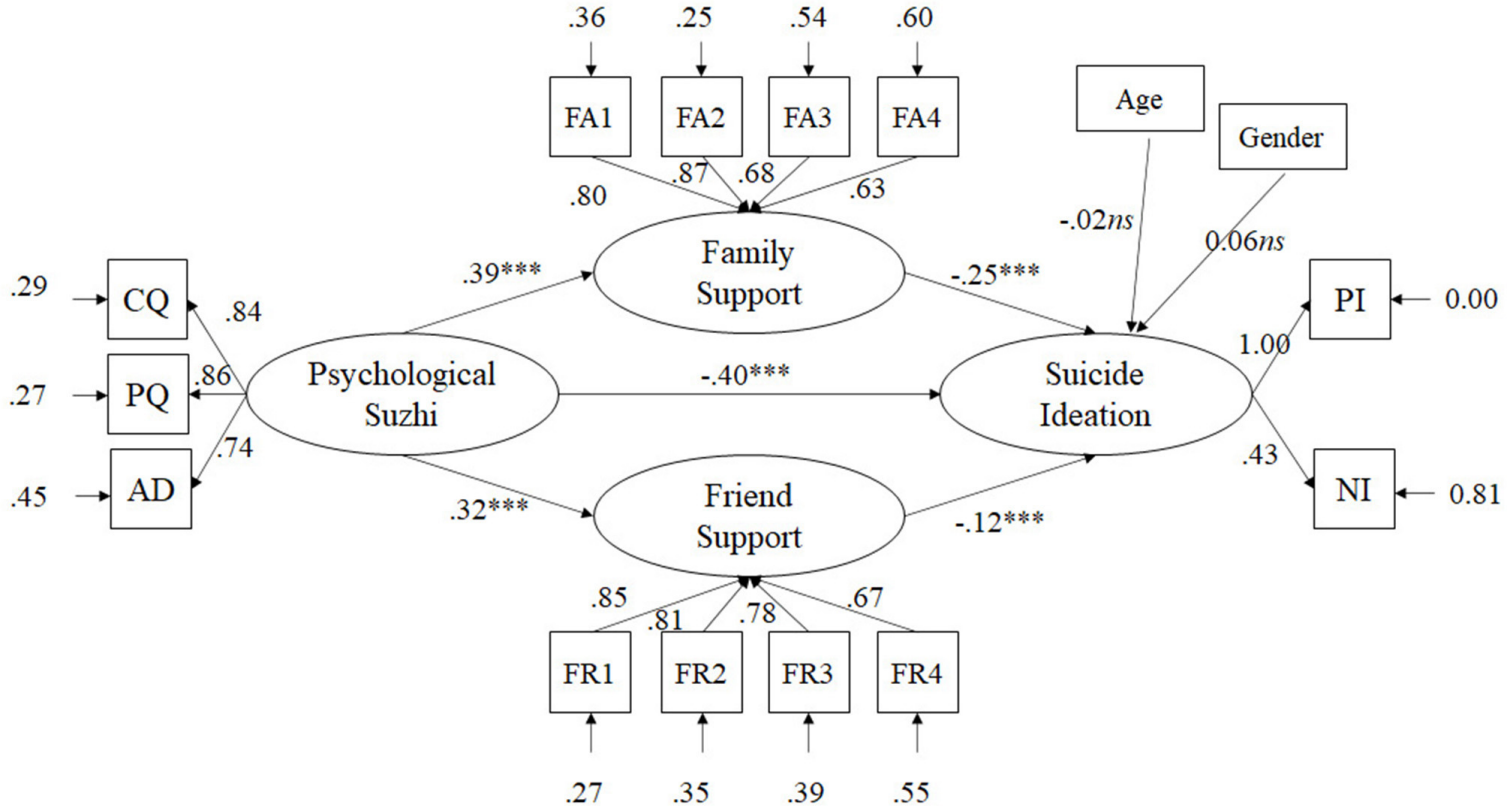
Structural equation model with standardized parameters estimates: psychological *suzhi* and suicide ideation. CQ, cognitive quality; PQ, personality quality; AD, adaptability; FR, friend support; FR1–FR4, four parcels of friend support; FA, family support; FA1–FA4, four parcels of family support. ***p < 0.001.

The standardized path coefficients among the main variables are presented in Figure 1. Psychological *suzhi* significantly related to suicide ideation (β = −0.40), supporting Hypothesis 1. The path coefficients between psychological *suzhi* and friend support (β = 0.32) and family support (β = 0.39) were both significant, supporting Hypothesis 2. Moreover, paths to suicide ideation from friend support (β = −0.12) and family support (β = −0.25) were both significant in the predicted directions. Therefore, friend support and family support mediated the association between psychological *suzhi* and suicide ideation; Hypothesis 3 was supported.

We subsequently used model constraint command of Mplus to create auxiliary variables and used bootstrapping in order to compare the mediation effects (Table 2). Psychological *suzhi*'s indirect correlation with suicide ideation was significantly stronger via family support than via friend support [β = −0.074, SE = 0.028, *p* < 0.01, 95% CI = (−0.025, −0.137)].

**Table 2:** Standardized indirect effects from psychological *suzhi* to suicide ideation.

**Indirect effect**	**β (standardized indirect effect)**	**SE**	***p***	**95% CI standardized indirect effect**
Sum of indirect	−0.134	0.019	<0.001	−0.174, −0.100
Via family support	−0.097	0.017	<0.001	−0.137, −0.067
Via friend support	−0.037	0.011	<0.01	−0.061, −0.017

## Discussion

In this research, we examined the relations of psychological *suzhi* and suicide ideation with the effect of family support and friend support as mediators. Our study findings showed that adolescents' psychological *suzhi*, family support, and friend support were significantly positively correlated with each other, and the three were significantly negatively linked with suicide ideation, which supports our Hypothesis 1 and Hypothesis 2. Consistent with prior research, whether it was objective social support or perceived social support, psychological *suzhi* had a good predictive effect (Zhang and Zhang, 2019; Zhang et al., 2019). Additionally, prior studies showed that perceived social support (including family support and friend support) could significantly negatively predict individuals' problem behaviors or even suicide ideation (Pace and Zappulla, 2013; Corbitt-Hall et al., 2018). Although there has not been any research investigating the relationship between psychological *suzhi* and suicide ideation, a follow-up study on Chinese adolescents found that students' psychological *suzhi* had a positive effect on individuals' depression, loneliness, and friendship quality (Zhang et al., 2017). These above variables were closely related to the individual's suicide ideation. Therefore, as the core element of mental health (Liu G. Z. et al., 2017), psychological *suzhi* could also effectively predict individuals' suicide ideation. This result suggests that improving adolescents' psychological *suzhi* may make them feel more support from family and friends and show a lower level of suicide ideation.

Regarding our third hypothesis, we found that psychological *suzhi* could affect individuals' suicide ideation through parallel mediation effect of family support and friend support by our model. That is to say, high level of psychological *suzhi* not only could directly reduce the individuals' suicide ideation, but also could be achieved by enhancing the individuals' perception of support from family and friends. This result supported the notion that family support and friend support had an essential part in the study life of Chinese students, which were consistent with previous studies (Rehman et al., 2020). It reveals that the mental health of adolescents is inseparable from the support and help of family and friends. At the same time, it is also vital to improve their psychological suzhi so that they can better perceive the support of others.

Moreover, we further compared the impact of family support and friend support, and the result showed that family support and friend support partially mediated psychological *suzhi*'s effect on suicide ideation, and the indirect effect was significant stronger via family support than via friend support. However, a study on migrant children in China showed that active friend support had an important protective effect on the individual's emotional and behavioral adaptation, while the protective effect of family support is only reflected in the field of behavioral adaptation (Wang et al., 2018). According to Poulin et al. (2012), in the background of Chinese culture, family support has a greater impact on the individuals' physical and mental health than friend support, but in Western countries, the result is the opposite. A possible explanation for this result is that teenagers are mainly in school life, and the connection between peer relationship and the daily emotional changes of adolescents is closer, but the parental rearing styles, behavior, and attitude toward their children have more important influence on serious problem behaviors such as suicide and crime. This is backed up by some studies. For example, family parenting had a significant impact on the personality traits in juvenile delinquents, and peer relationship only played a moderating role in some ways (Peng et al., 2013). Other studies pointed out that when it comes to stable and long-lasting emotions such as subjective well-being, compared with friend support, the predictive effect of the individuals' own behavior is more obvious (Traylor et al., 2016).

## Limitations and Future Directions

Some limitations of this research should also be noted, which can provide some suggestions and guidance for future research. First, we used a cross-sectional design to test our hypotheses, thereby the current results are preliminary, and we try to use longitudinal or experimental design in the future. Second, this study was conducted in Chinese students; the cultural discrepancies may result in different consequences. Future research can further compare the results under different cultures. Finally, perceived social support may not be consistent with the actual social support, and comparing their impact is also a meaningful topic.

## Conclusions

In conclusion, this study investigates how adolescents' psychological *suzhi* influences suicide ideation. Specifically, we found that adolescents' psychological *suzhi* is negatively related to suicide ideation, and family support and friend support partially mediate this effect. Meanwhile, compared with friend support, family support plays a more important role in reducing adolescents' suicide ideation.

## Data Availability Statement

The raw data supporting the conclusions of this article will be made available by the authors, without undue reservation.

## Ethics Statement

The studies involving human participants were reviewed and approved by the Research Ethics Committee of Southwest University approved the study. Written informed consent to participate in this study was provided by the participants' legal guardian/next of kin.

## Author Contributions

On the basis of reading the relevant literature, ZZ put forward the research questions and the solution, and assisted in study design, data collection, and writing work. At the same time, according to the teachers' and cooperative researchers' recommendations and comments have been amended to formally form the final submission manuscript. DZ was mainly responsible for the supervision and guidance of the entire research process and providing financial support. WT and GL were mainly responsible for modifying and providing guidance to this paper, played an important role in the successful writing of the paper. All of the authors participated in the final approval of the version to be published and agreed to be accountable for all aspects of the work.

## Conflict of Interest

The authors declare that the research was conducted in the absence of any commercial or financial relationships that could be construed as a potential conflict of interest.
