# Supplementary material for: The Effect of Psychological Suzhi on Suicide Ideation in Chinese Adolescents: The Mediating Role of Family Support and Friend Support
Source: Front Psychol. 2021 Feb 11;11:632274. doi: 10.3389/fpsyg.2020.632274 (PMC7905087; doi:10.3389/fpsyg.2020.632274)
Supplement: Supplementary file 1 [file Table_1.docx]

**Supplementary materials**

Psychological *Suzhi* Questionnaire for Middle School Students: 1 (totally disagree) to 5 (totally agree).

1. I always have explicit pathways when doing exercises.
2. I always press myself to complete what I should do.
3. I am a popular person.
4. I can arrange and control my entertainment.
5. I am interested in new course content.
6. I keep good relationships with my teachers.
7. I usually do my things on my own.
8. I always do well in group activities.
9. I always set appropriate goals and plans of learning.
10. I can often effectively resolve the embarrassment.
11. When solving a problem, I usually have a sense of what kind of theorems and methods I have used.
12. I can face the frustrations in life bravely and never give up.
13. I can calmly response to the critical condition.
14. I never put off until tomorrow what I can do today.
15. I always build up associations between previous knowledge and the new.
16. In the learning process, I can timely initiative to adjust the way of learning.
17. I always adhere to my plan.
18. I get on well with my classmates.
19. I can solve problems by myself.
20. I can find a variety of resources to solve learning puzzles.
21. I can blend into my current surroundings.
22. I am always strict with myself.
23. I often organize or attend many class activities.
24. I can select appropriate methods based on tasks.
